# In Vivo Effects of Methionine Sulfoxide Reductase Deficiency in *Drosophila melanogaster*

**DOI:** 10.3390/antiox7110155

**Published:** 2018-11-01

**Authors:** Lindsay Bruce, Diana Singkornrat, Kelsey Wilson, William Hausman, Kelli Robbins, Lingxi Huang, Katie Foss, David Binninger

**Affiliations:** 1 Department of Biological Sciences, Charles E Schmidt College of Science, Florida Atlantic University, Boca Raton, FL 33431, USA; lindsay.e.bruce@gmail.com (L.B.); dsingkor@my.fau.edu (D.S.); kelseywilson2015@fau.edu (K.W.); Billy@dnalabsinternational.com (W.H.); krobbin2@fau.edu (K.R.); goofy999@bellsouth.net (L.H.); lhuang2012@my.fau.edu (K.F.); 2 Center for Molecular Biology and Biotechnology, Charles E Schmidt College of Science, Florida Atlantic University, Boca Raton, FL 33431, USA

**Keywords:** aging, repair, methionine sulfoxide reductase, *Drosophila*, oxidoreductases, oxidative stress, physiological function

## Abstract

The deleterious alteration of protein structure and function due to the oxidation of methionine residues has been studied extensively in age-associated neurodegenerative disorders such as Alzheimer’s and Parkinson’s Disease. Methionine sulfoxide reductases (MSR) have three well-characterized biological functions. The most commonly studied function is the reduction of oxidized methionine residues back into functional methionine thus, often restoring biological function to proteins. Previous studies have successfully overexpressed and silenced MSR activity in numerous model organisms correlating its activity to longevity and oxidative stress. In the present study, we have characterized in vivo effects of MSR deficiency in *Drosophila*. Interestingly, we found no significant phenotype in animals lacking either methionine sulfoxide reductase A (MSRA) or methionine sulfoxide reductase B (MSRB). However, *Drosophila* lacking any known MSR activity exhibited a prolonged larval third instar development and a shortened lifespan. These data suggest an essential role of MSR in key biological processes.

## 1. Introduction

Free radical production is an unavoidable consequence of aerobic respiration and contributes to the aging process as well as certain neurodegenerative diseases (reviewed in references [1,2,3,4,5,6]). Collectively known as reactive oxygen species (ROS), these potent sources of oxidative stress are primarily produced through cellular respiration in the mitochondria, although ROS are also produced from various cellular oxidases as well as extracellular sources such as ultraviolet light, radiation and toxins found in the environment. Oxidative damage to macromolecules is now generally accepted as a major factor in the etiology of age-related diseases and the aging process itself, as first postulated by Harman [7]. It is also established that organisms have developed protective mechanisms against high levels of ROS [8]. However, the level of ROS in cells must be carefully regulated, since low levels of ROS can function as signaling molecules that help protect the cells against oxidative damage [9,10,11], whereas excessive amounts can lead to cell death. These protective mechanisms include intracellular antioxidants such as glutathione, enzymes that destroy the ROS before they can do damage, including catalase and superoxide dismutase and mechanisms to repair damage to macromolecules, such as the extensively studied DNA repair systems [8]. More recently there has been special interest in a protein repair system, the methionine sulfoxide reductase (MSR) family that can reverse damage to proteins due to oxidation of methionine residues in proteins to methionine sulfoxide (met-(o)). The MSR system can reduce the met-(o) back to methionine stereospecifically since met-(o) formed by chemical oxidation is a mixture of the R (met-R-(o)) and S (met-S-(o)) epimers. The two most studied MSR proteins, referred to as methionine sulfoxide reductase A (MSRA) and methionine sulfoxide reductase B (MSRB), specifically reduce the S and R epimers, respectively [12].

Initially, it was assumed that the only function of the MSR system was to repair oxidative damage to proteins. Weissbach, Brot and colleagues originally showed that inactive *Escherichia coli* ribosomal protein L12 has oxidized methionine residues [13] that could be reduced by MSRA [14]. It is now known that the MSR enzymes can also function as part of an ROS scavenger system as first suggested by Levine and coworkers [15]. In this process, methionine residues in proteins can function as catalytic antioxidants since each round of ROS oxidation of a methionine residue and reduction of the met-(o) by an MSR enzyme, will destroy one equivalent of the ROS [12,16,17]. In addition, a third, and potentially important role of the MSR system is the regulation of protein activity. The function of ion-channels within the cell membrane can be altered depending on the oxidation/reduction state of methionine residues [18,19,20,21]. More recently, it has been reported that oxidation and reduction of specific methionine residues in actin can regulate the interconversion of F-actin to G-actin. Mical, a known actin-binding protein, is a member of a family of flavin monooxygenases that can catalyze the stereospecific oxidation of two methionine residues in F-actin to met-R-(o), leading to the disassembly of the F-actin and the formation of G-actin. MSRB can reduce the met-R-(o) residues in G-actin back to methionine, resulting in assembly of the actin fibers and the formation of F-actin [22]. 

The biological importance of the MSR system, especially MSRA, has been demonstrated in numerous studies. In summary, almost uniformly, knockout of *MSRA* leads to increased sensitivity to oxidative stress, which includes studies in an *MSRA* knockout mouse [23,24,25,26]. Where examined, there is also an increase in the levels of ROS in cells lacking MSRA. In contrast, cells with elevated levels of MSRA have lower levels of ROS, are more resistant to oxidative stress and elevated levels of MSRA can extend the lifespan of flies [27,28], worms [29] and yeast [30], but not mice [31]. 

All animals appear to have one *MSRA* gene, but mammals contain three *MSRB* genes. There is less known about the role of the MSRB enzymes. MSRB1 is a selenoprotein located in the cytoplasm and is the most abundant mammalian MSRB isoform. A complementary study to knocking out *MSRA* in mice involved feeding mice a selenium-deficient diet, which effectively created *MSRB1* knockout mice. Although the *MSRB1* knockout mice showed an increased sensitivity to oxidative stress, the phenotype was not nearly as severe as the *MSRA* knockout mice [32]. Overexpression of mammalian MSRB1 in *Drosophila* neurons caused an increased resistance to oxidative stress and starvation but did not affect lifespan or reproduction [33]. Also, overexpression of endogenous *Drosophila* MSRB was found to have no effect on lifespan but overexpression of mammalian MSRB3 did extend the lifespan of *Drosophila* [34]. 

Until the advent of the CRISPR-Cas9 system, a knockout of both *MSRA* and *MSRB* in mammals was a technically difficult feat involving the removal of four genes simultaneously and had not been reported. However, Levine et al., have utilized CRISPR-Cas9 to create a quadruple MSR knockout mouse from the *MSRA/MSRB1* knockout mouse, although the phenotype of this organism has yet to be fully elucidated [35]. *Drosophila* has only one *MSRB* gene, and this study, using *Drosophila melanogaster*, characterizes for the first time a genetic animal model lacking both MSRA and MSRB. 

## 2. Materials and Methods

### 2.1. Fly Stocks

All stocks were maintained on standard cornmeal agar medium (Genesee Scientific, El Cajon, USA) at 25 °C on a 12 h day/night cycle. Wild-type stock lines included yellow white (YW) and Biocore (courtesy of the R. Murphey and K. Dawson-Scully, respectively, both at Florida Atlantic University, Boca Raton, FL, USA). The Biocore strain (FlyBase ID FBal0033148) was originally obtained from the Bloomington Stock Center (Bloomington, IN, USA) as were the fly stocks containing P-elements in the *MSRA* and *MSRB* loci. The line with the P-element (EY05753) insertion in *MSRA* was numbered 16671 (Genotype: y[1] w[67c23]; P{w[+mC] y[+mDint2] = EPgy2}Eip71CD[EY05753]). The line with the P-element (EP3340) in MSRB was numbered 17116 (Genotype: w^1118^; P{w[+mC] = EP}SelR[EP3340]/TM6B, Tb[1]). Stock 7956 (Genotype: w^1118^; Df(3R)Exel7305/TM6B, Tb^1^) was also obtained from the Bloomington Stock Center. The transposase line (Genotype: w; Gla/CyΔ2-3; +) was obtained from R. Murphey (Florida Atlantic University, Boca Raton, FL, USA).

### 2.2. Generation of Deletion Mutations in MSR Loci

Imprecise excision of P-elements in strains 16671 and 17116 (described above) was obtained through the introduction of transposase (w; Gla/CyΔ2-3; +). The *MSRA* and *MSRB* loci are both located on the third chromosome. Genetic recombination between the MSRA^∆/∆^MSRB^+/+^ and MSRA^+/+^MSRB^∆/∆^ lines yielded the MSRA^∆/∆^MSRB^∆/∆^ line. The four genotypes were backcrossed multiple times to YW to minimize genetic differences in the progeny. A detailed discussion of how the genetic strains used in this study were created and the rationale for their selection is given in the Supplementary Materials.

### 2.3. Reverse Transcription Polymerase Chain Reaction (RT-PCR)

cDNA synthesis by reverse transcription used standard methods. More specifically, a 25 µL reaction contained 5 µL of 5× Moloney murine leukemia virus (M-MLV) Buffer (Promega, Madison, WI, USA), 100 µM each of dATP, dCTP, dGTP and dTTP (dNTP) (Qiagen, Germantown, MD, USA), 1 µL of M-MLV Reverse Transcriptase (Promega; 200 U/µL), 1 µL RNAse Inhibitor (Bioline; Memphis, TN, USA, 40 U/µL), 250 ng of random primers (Promega) and 1 µg total RNA. The cDNA was then used as a template for amplification by PCR using standard techniques. For MSRA, the forward primer was TGAGCACCGTTCGCAATGAA while the reverse primer was ATGCAGATTCGGCCATGTCA. For MSRB, the forward primer was AATCTCGGACCAGCAGCGACTATG while the reverse primer was CTCGAGATCACTGCTGGGCAATGGG. For rp49, the forward primer was TGACCATCCGCCCAGCATACA while the reverse primer was TCTCGCCGCAGTAAAC. For each PCR amplification, 40 cycles were used after an initial denaturation at 94 °C for 5 min. The annealing temperatures were as follows: 52 °C for MSRA; 59 °C for MSRB; 50 °C for rp49.

### 2.4. Western Blotting

Protein was extracted from approximately 20 adult flies in a buffer containing 150 µL of 150 mM NaCl in 2.5 mM Tris·Cl, pH 7.5. Samples were pestle homogenized and centrifuged at 16,000× *g* for 20 min at 4 °C to obtain the supernatant. Protein concentration was determined using the Bradford assay [36]. Protein (20 µg) was used for each sample on the Western blot. The 15% polyacrylamide gels were run at 140 volts for 1.5 h. Protein was transferred to polyvinylidene difluoride (PVDF) membrane (Immobilon-P, MilliporeSigma, Burlington, VT, USA) at 15 volts for 60 min using a semi-dry transfer cell (Trans-Blot SD, Bio-Rad, Hercules, CA, USA). The membrane was blocked in 5% powdered skim milk in Tris-Tween-Buffer-Saline (TTBS) for 90 min at room temperature. Primary polyclonal antibodies from rabbit using purified recombinant *Drosophila* MSRA and MSRB proteins were prepared by Covance (San Diego, USA). The MSRA primary antibody was diluted 1:500 and the MSRB primary antibody was diluted 1:800. A primary antibody recognizing alpha tubulin (MilliporeSigma, Burlington, VT, USA) was diluted 1:250 for use as an internal loading standard. Goat anti-rabbit conjugated with horseradish peroxidase (HRP) was used as a secondary antibody at a 1:1000 dilution (catalog number AP132P, MilliporeSigma, Burlington, VT, USA). The detection reagent was Immun-Star HRP Chemiluminescent Kit (Cat# 170-5040, Bio-Rad, Hercules, CA, USA). Western blots were visualized using the VersaDoc imaging system (Bio-Rad, Hercules, CA, USA) and the G:Box chemiluminescence system (Syngene, Frederick, MD, USA).

### 2.5. Lifespan

The lifespan assay was performed on standard cornmeal agar medium in a 25 °C incubator on a 12 h day/night cycle. Flies were reared in bottles, which were cleared approximately 10–14 days after the bottles were started. Clearing day was considered Day 1. Male flies were collected on Day 5 and sorted in groups of 10 into vials containing 5 mL of medium. New food was provided twice weekly and survival was scored three times weekly. Survival data were analyzed using Prism (GraphPad, La Jolla, CA, USA). A normal fly death was counted as a 1, while abnormalities were scored as 0. Survival curves were statistically analyzed via the Log-rank test. Mean lifespan ± standard error of the mean (SEM) was also calculated for each genotype and analyzed between subjects using one-way analysis of variance (ANOVA) with Tukey’s Post Hoc test.

### 2.6. Oxidative Stress Assay

The oxidative stress assay was performed as described in the lifespan assay except that the medium was supplemented where indicated with a final concentration of 2 mM methyl viologen dichloride (Thermo Fisher Scientific, Waltham, MA, USA), known more commonly as the herbicide paraquat.

### 2.7. Protein Carbonyl Assays

The original colorimetric 2,4-dinitrophenylhydrazine (DNPH) assay was developed by Levine et al. [37,38]. Our modified version involved protein extracted from approximately 100 adult flies in 1.2 mL of buffer containing 150 mM NaCl in 2.5mM Tris·Cl, pH 7.2. Samples were pestle homogenized and centrifuged at 16,000× *g* for 20 min at 4 °C to obtain 1 mL of the supernatant. The supernatant was divided into two 500 μL samples. Each was treated with equal volumes of 20% trichloroacetic acid (TCA), vortexed, incubated on ice for 10 min followed by centrifugation at 2500× *g* for 5 min at 4 °C. The supernatant was discarded and each sample was washed in 10% TCA, vortexed, incubated on ice for 10 min, and then centrifuged at 2500× *g* for 5 min at 4 °C. The supernatant was discarded and one portion of the sample was resuspended in 600 μL 10 mM DNPH in 2M HCl, while the other portion was resuspended in 600 μL 2M HCl. The samples were sonicated (XL2000 Series, Misonix, Farmingdale, NY, USA) for 5 s and kept in the dark at room temperature for 1 h, vortexing every 15 min. After incubation, another TCA precipitation was performed by adding 600 μL of 20% TCA to each sample followed by incubation on ice for 10 min before centrifuging at 2500× *g* for 10 min at 4 °C. The supernatant was discarded and the protein pellet was washed with 1 mL of 1:1 ethanol/ethyl acetate, sonicated, and centrifuged at 1000× *g* for 10 min at room temperature. This wash-sonication-centrifugation protocol was repeated three more times. The protein pellet was then resuspended in 650 μL of 6M guanidine-HCl, vortexed, and incubated at 37 °C for 5 min to allow the pellet to fully solubilize. The samples were centrifuged at 1000× *g* for 15 min at room temperature and the majority of the supernatant removed without touching the bottom or sides of the microcentrifuge tube. The absorbance of both the DNPH treated and HCl control samples was measured at 370 nm. Samples of oxidized bovine serum albumin (BSA) at three different concentrations were processed in the same manner to ensure that the procedure was working properly. A Bradford assay [36] was used to normalize protein concentration using the Protein Assay Dye Reagent (catalog #500-0006; Bio-Rad, Hercules, CA, USA) and the SpectraMax plate reader (Molecular Devices, San Jose, CA, USA) using BSA to generate a standard curve. Final standardized measurements of protein carbonyl levels were statistically analyzed using a one-way between subjects ANOVA (Prism).

### 2.8. Larval Development

**Pre-laying for freshly fertilized eggs.** To synchronize egg laying, approximately 50–100 flies were allowed to lay eggs in a small amount of yeast paste on grape juice agar medium (30% grape juice, 0.6% sucrose, 2.4% agar and 0.8 mg/mL methylparaben) for 1 h at 25 °C. The eggs were discarded and the process repeated a second time. Eggs from the second pre-lay were also discarded. Females were then transferred to fresh medium that was designated time 0 h for larval development.

**Determination of time from egg laying to pupariation and eclosion of adult flies.** After performing a pre-lay, eggs were transferred, using a toothpick, to a food vial containing approximately 8 mL of standard cornmeal food at 20 eggs per vial. The vials were kept in a 25 °C incubator and monitored twice a day for 11 days. The pupariation stage was identified by lack of movement, hardening of the cuticle, and eversion of the spiracles. These were counted and marked with a Sharpie pen to assure old puparia were not counted at multiple time points. The adult stage was identified by counting the number of adult flies present at the time evaluated. The duration of the pupal stage was determined by subtracting the time at eclosion from the time at pupariation. Five trials were performed for each line (*n* = 5).

**Determination of time from egg laying to first instar larva.** After performing a pre-lay, 20 fresh eggs were placed on a grape juice agar plate. Four rows were set up containing five eggs each with even spacing. The grape agar plates containing the eggs remained in the 25 °C incubator for the duration of the experiment aside from evaluating them. The number of larvae that emerged from the eggs was counted at 21, 25, and 29 h. Five trials were performed for each line (*n* = 5).

**Determination of time from egg laying to the second instar stage.** After performing a pre-lay, 20 fresh eggs were placed in standard cornmeal vials which were maintained in the 25 °C incubator. To evaluate this stage, larvae were washed out of the media using phosphate buffered saline (PBS) at several time points. Once the larvae were washed out of the food, they were transferred to a slide using a paintbrush. Once on the slide, they were viewed under a microscope at 40× magnification and the configuration of the anterior spiracles and mouth hooks were evaluated. The appearance of the anterior spiracles was used to determine the developmental stage. If the anterior spiracles presented as projections at the end of the tracheal trunk then it was counted as a second instar. The larvae that had not yet reached the second instar stage were placed in a new cornmeal vial for evaluation during the next time point. This stage was evaluated at 46, 50, and 54 h. Five trials were performed for each line (*n* = 5).

**Determination of time from egg laying to the third instar.** After performing a pre-lay, 20 fresh eggs were placed in standard cornmeal vials which were maintained in the 25 °C incubator. For this stage, anterior spiracles and mouth hooks of the larvae were evaluated by examination using a microscope at 40× magnification as described above. If the anterior spiracles presented as branched projections at the end of the tracheal trunk then it was counted as a third instar. The larvae that had not yet reached the third instar stage were placed in a new cornmeal vial for evaluation during the next time point. This stage was evaluated at 71, 75, 79, and 83 h. Five trials were performed for each line (*n* = 5).

**Determination of time from egg laying to the wandering third instar.** After performing a pre-lay, 20 fresh eggs were placed in standard cornmeal vials that were maintained at 25 °C. The vials were evaluated twice a day between days 4 and 8. Wandering third instars were identified by emergence from the food. Five trials were performed for each line (*n* = 5). Duration of the wandering third instar was calculated by subtracting the time of pupariation from the start time of the wandering third instar. Exact durations of the wandering third instar were not able to be obtained.

### 2.9. Determination of Wet and Dry Larval Mass

After performing a pre-lay, 30 fresh eggs were placed in standard cornmeal vials which were maintained at 25 °C. At the indicated time after egg-laying (72 and 96 h), the larvae were washed out of the food and sorted by sex. The sex was determined by the size of the gonads, which can be found in the fifth abdominal segment and present as transparent oval-shaped bodies within the fat. Males have significantly larger gonads than the females. After the larvae were sexed, 10 males and 10 females were placed in respective microcentrifuge tubes. The larvae were frozen at −80 °C for approximately 1 h. The wet mass samples were determined using an analytical balance. The larvae were then transferred back into the microcentrifuge tube and placed in a 70 °C oven for 24 h with the lids open. The dry mass of the larvae was then measured using an analytical balance. The total mass of the sample was divided by the number of larvae used (10) to determine the body mass per larva. Five trials were performed for each line (*n* = 5).

The wet and dry masses of wandering third instar larvae (identified as larvae climbing out of the food), newly eclosed flies (distinguished by their light pigment and absence of wing expansion) and 6–7 day old adult flies (sexed by morphology of the gonads and the presence/absence of sex combs) were determined in a similar manner.

### 2.10. Mouth Hook Contraction Assay

Third instar larvae (confirmed as described above) were transferred to small Petri plates containing 10% glucose in 2.3% agar. Larvae were given one minute to acclimate to the medium and then mouth hook contractions were visually quantified using a hand-held mechanical counter while viewing with a dissection microscope.

### 2.11. Measurement of ^32^P-Labeled Food Intake

After performing a pre-lay as described above, eggs were transferred via toothpick into food vials containing approximately 10 mL of standard cornmeal food. Vials were maintained in a 25 °C incubator on 12 h dark/light cycle. Larvae were then extracted from the standard cornmeal food 84 h after egg laying using 1× PBS. Larvae were sorted based on spiracle confirmation to confirm the third instar stage. Three to eight larvae were placed into each vial, containing 3 mL of standard cornmeal food supplemented with 5 µCi ^32^P-dCTP and allowed to feed for 5.5 h at 25 °C. Then larvae were extracted from the food utilizing 1× PBS. Larvae were promptly washed three times with 1× PBS to minimize background due to food adhering to larval bodies. This step was performed rapidly to minimize chances of larval excretion. The larvae were then placed in 5 mL of scintillation fluid and the level of radiolabel was measured in a multipurpose scintillation counter. Background levels of radiolabel were determined using a 200 µL aliquot of the third wash.

### 2.12. Statistics

The statistical significance of the developmental checkpoint assays was evaluated using Prism software. Average time when the development checkpoints were reached was used for each trial. *N* = the number of vials/plates per strain. Both ANOVA and Tukey’s tests were performed for each experiment. The statistical significance of the mass assays was evaluated using Prism software. Average mass at specific checkpoints was used for each trial. *N* = the number of vials used per strain. Both ANOVA and Tukey’s tests were performed for each experiment. The results of the mouth hook contraction assay were analyzed using Prism software. *N* = the number of trials; each trial was one larva, with six trials for each of the WT60 and AB46 lines. Statistical tests performed were *t*-test and 2-way ANOVA.

## 3. Results

### 3.1. Characterization of Lines Having Complete Loss-Of-Function (Null) Alleles of MSRA and MSRB

Imprecise P-element transposon excision was used to create deletions in the *MSRA* and *MSRB* loci. The 1.5 kb deletion in *MSRA* (*MSRA^∆/∆^MSRB^+/+^*) starts 300 bp upstream of the transcription start site and extends 1172 bp into the transcribed region, terminating in Exon 2. The deletion includes the entire 5’ UTR and a portion of the open reading frame. The 2.5 kb deletion in *MSRB* (*MSRA^+/+^MSRB^∆/∆^*) extends from 364 bp upstream of the transcription start site to 2163 bp into the transcribed region. The deletion removes the first three exons, which also includes a portion of the open reading frame. Both *MSR* loci are located on chromosome 3, so genetic recombination was used to recover the double *MSR* deletion (*MSRA^∆/∆^MSRB^∆/∆^*).

**Figure 1:**
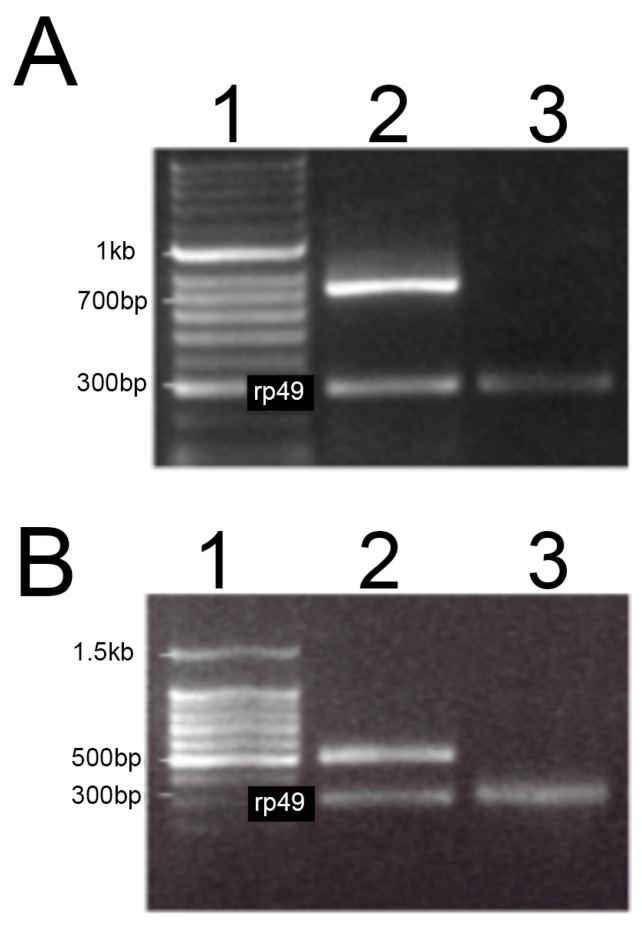
Reverse-transcription polymerase chain reaction (RT-PCR) amplification of methionine sulfoxide reductase (*MSR*) null mutants. Total RNA was used for cDNA synthesis using standard techniques. The cDNA was used as a template for PCR amplification using primers capable of amplifying all isoforms of transcripts of methionine sulfoxide reductase A (*MSRA;* Panel **A**) or methionine sulfoxide reductase B (*MSRB*; Panel **B**) as described in the text. The PCR amplicons were then resolved by agarose gel electrophoresis. Lane 1 in both panels shows DNA size markers (Promega). PCR product in Lane 2 utilized cDNA derived from RNA isolated from the yellow white (YW) strain, which has wild-type alleles for both *MSR* genes. Lane 3 utilized cDNA derived from RNA isolated from strain A63, an *MSRA* deletion (Panel **A**) or B54, an *MSRB* deletion (Panel **B**). The 300 bp PCR product labeled rp49 (ribosomal protein 49) served as the internal loading control.

**Figure 2:**
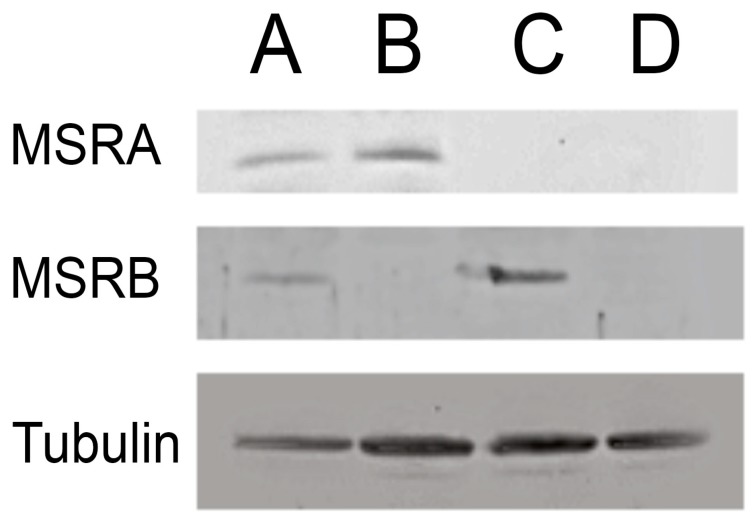
Western blot analysis of methionine sulfoxide reductase (MSR) mutant strains. Protein extracts were used for Western blot analysis following standard procedures. The primary antibody used for each blot is indicated at the left edge. Lane A is the wild-type strain (WT60), Lane B is the *MSRB* deletion (B54) line, Lane C is the *MSRA* deletion (A63) strain and lane D is an *MSRA/MSRB* double deletion (AB46) line. α-tubulin was used as a loading control.

No detectable transcripts were observed in either the *MSRA* or *MSRB* deletion lines using reverse transcription PCR (Figure 1). Western blotting confirmed that the deletions exhibited undetectable levels of protein from the corresponding gene (Figure 2).

Table 1 summarizes the strains that were used for the results presented here. They are sibling lines that were backcrossed for multiple generations to a well-characterized wildtype lab strain (YW) to minimize background genetic variation among the four strains.

### 3.2. MSR Deficiency Shortens Lifespan

**Figure 3:**
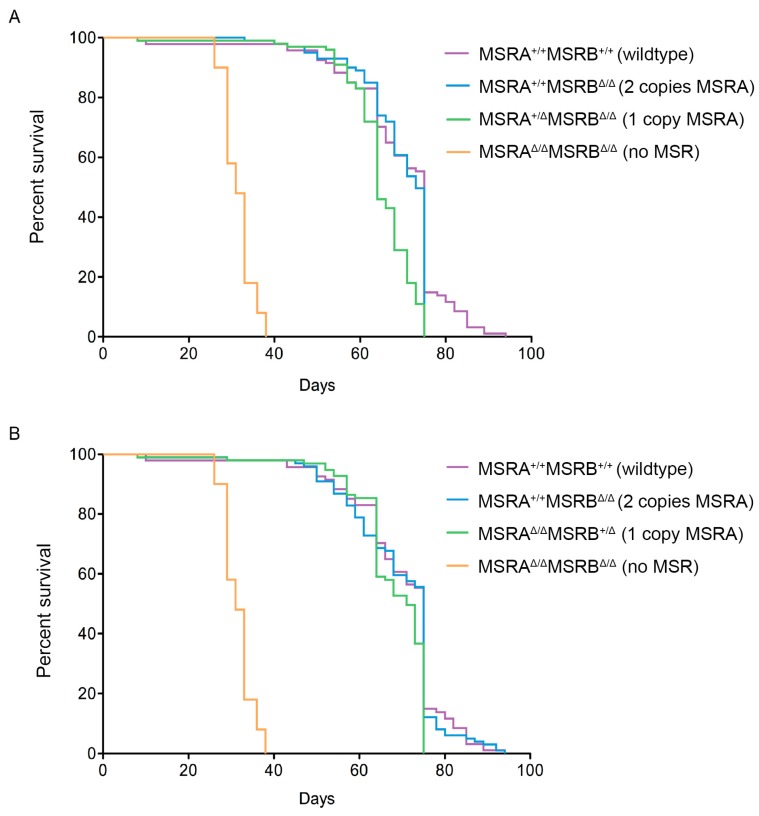
Lifespan of *Drosophila* is shortened in the absence of MSR activity. The experimental design is described in the text. The four main fly strains were used (WT60—wild-type, A63—two copies *MSRB*, B54—two copies *MSRA*, and AB46—no functional *MSR* loci; Table 1). The lines containing a single copy of *MSRA* were made by crossing AB46 to B54 and collecting the progeny. The lines containing a single copy of *MSRB* were made by crossing AB46 to A63 and collecting the progeny. The log-rank test was used to determine significant differences between the survival curves. One wild-type allele of either the *MSRA* (Panel **A**) or *MSRB* (Panel **B**) gene is sufficient to rescue the lifespan of the *MSR* double-deletion mutant to nearly that of the wild-type (*p* < 0.001).

The strain having homozygous *MSRA* deletions (A63) and the strain having homozygous *MSRB* deletions (B54) had lifespan curves that were nearly identical to the wild-type (WT60) (Figure 3). However, homozygous loss of both *MSR* loci (AB46) significantly decreased lifespan compared to both of the homozygous single *MSR* deletion lines and the wild-type control. A single copy of the wild-type allele of either *MSRA* or *MSRB* was sufficient to almost completely rescue the lifespan (Figure 3).

### 3.3. Protein Oxidation

**Figure 4:**
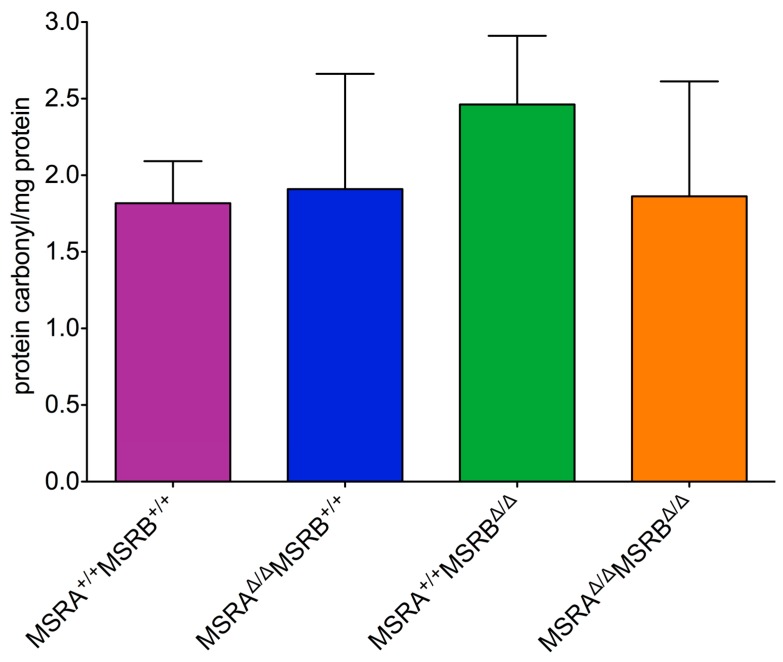
MSR deficiency does not increase sensitivity to oxidative stress. Protein extracts were treated with 2,4-dinitrophenylhydrozine and analyzed via spectrophotometry as described in the text. An oxidized BSA standard was used as a control. No significant differences between genotypes was observed (one-way ANOVA, F[3,12] = 0.2597, *p* = 0.8530).

Protein oxidation is typically elevated in organisms experiencing oxidative stress and would be expected to be elevated in the MSR mutant strains since, as described above, the MSR system has been shown to protect organisms against chemical oxidation by ROS and has been shown to affect ROS levels in cell culture. Therefore, an increase in the levels of protein oxidation (as measured by protein carbonyl formation) was a predicted result of MSR-deficiency. Unexpectedly, there was no significant difference in the amount of protein oxidation between the wild-type line and any of the MSR mutants including the strain that was homozygous for deletions in both *MSR* loci (Figure 4). The data indicated that under standard growth conditions there is not a significantly elevated level of oxidative damage in the MSR mutant strains.

### 3.4. MSR Mutants Are Not More Sensitive to Oxidative Stress

**Figure 5:**
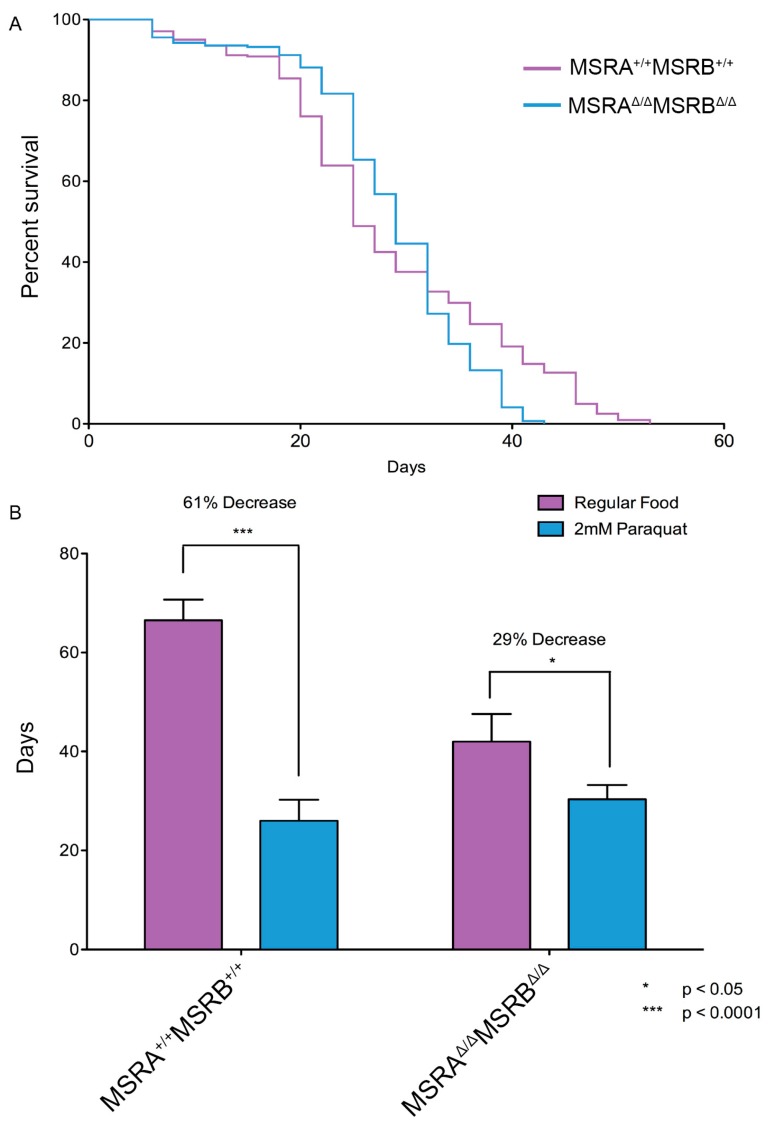
Exposure to oxidative stress minimizes lifespan differences. (**A**) Survival of the MSRA^∆/∆^MSRB^∆/∆^ line was significantly better than the wild-type in the presence of 2 mM paraquat (log-rank test; *p* = 0.0261). (**B**) The median lifespan of the wild-type and MSRA^∆/∆^MSRB^∆/∆^ lines were not significantly different on medium containing 2 mM paraquat (compare both blue columns, *t*-test, *p* = 0.1916). The change in the median lifespan when the medium contained 2 mM paraquat was substantially greater in the wild-type flies (61% decrease, *t*-test, *p* = 0.0006) than the MSRA^∆/∆^MSRB^∆/∆^ mutants (29% decrease, *t*-test, *p* = 0.0322).

Perhaps the protective effect of the MSR enzymes would only be observed when the animals were under environmental oxidative stress. To test this hypothesis, the MSR deletion mutants were exposed to paraquat (methyl viologen dichloride), a herbicide that is highly toxic because it generates intracellular free radicals. The *MSR* double deletion mutant was expected to be hypersensitive to paraquat and this effect would exacerbate the difference in lifespans seen under normal conditions. However, as shown in Figure 5A, the lifespan curves for the wild-type and *MSR* double deletion mutant were quite similar when the medium contained 2 mM paraquat. In fact, the proportional decline in median lifespan in the presence of 2 mM paraquat was significantly greater for the wild-type strain (Figure 5B).

### 3.5. MSR Deficiency Delays Third Instar Larval Development

While completing the lifespan experiments, we noticed that the double *MSR* mutant strain (AB46) took nearly an extra day to reach eclosion. More careful examination revealed that neither of the *MSR* single-mutants (A63 and B54) showed any difference in the rate of development from the first instar to eclosion when compared to the wild-type. However, the strain that was homozygous for deletions in both *MSR* loci (AB46) took nearly 20 h longer to reach eclosion. Furthermore, this delay was confined to the early/mid third instar when the larvae were vigorously feeding on the culture medium. There was no significant difference in the duration of the first or second instar or the period from the wandering third instar (larvae are crawling out of the food in preparation for pupation) to eclosion among any of the four genotypes. The data are summarized in Table 2.

### 3.6. MSR-Deficient Larvae Have Significantly Less Mass at 96 h after Egg Laying

Despite the double *MSR* deletion larvae (AB46) having a significantly longer duration of the early-mid third instar stage, the wet masses of female larvae remained the same near the beginning third instar/late second instar and the initiation of the wandering third instar. However, at 96 h, commonly designated mid-third instar, the mass of the double *MSR* mutant larvae (AB46) was about 46% less than that of the wild-type (WT60). There were no significant differences in the wet masses of females of all four genotypes for the wandering third instar larvae, newly eclosed adult flies and 6-day old adult flies. The data are summarized in Table 3. Male larvae showed the same trend as the female larvae, though overall, they were smaller (data not shown). The experiments were repeated to determine dry masses, which showed a similar result (data not shown). In summary, there were no significant differences in wet or dry masses for either male or female larvae in *MSRA* and *MSRB* single-mutant larvae compared to wild-type at any stage from first instar larvae to 6-day old adult flies. However, the larvae having homozygous deletions in both *MSR* loci (AB46) had a markedly longer mid-third instar period of about 21 h. All other phases in growth were not significantly different compared to wild-type.

### 3.7. MSR-Deficient Larvae Consume Food at a Slower Rate

**Figure 6:**
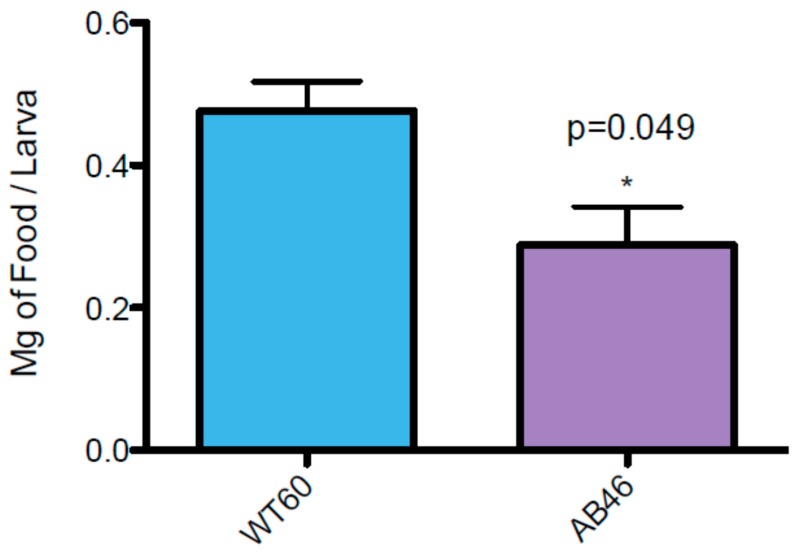
MSR-deficient larvae consume food at a slower rate. Wild-type (WT60) and MSR-deficient (AB46) larvae were maintained on standard cornmeal food containing a ^32^P label for 5.5 h. After washing larvae to remove excess radiolabel, the ^32^P content of the larvae was determined. The data are based on three trials per genotype. Error bars show SEM (*t*-test; * *p* = 0.049).

**Figure 7:**
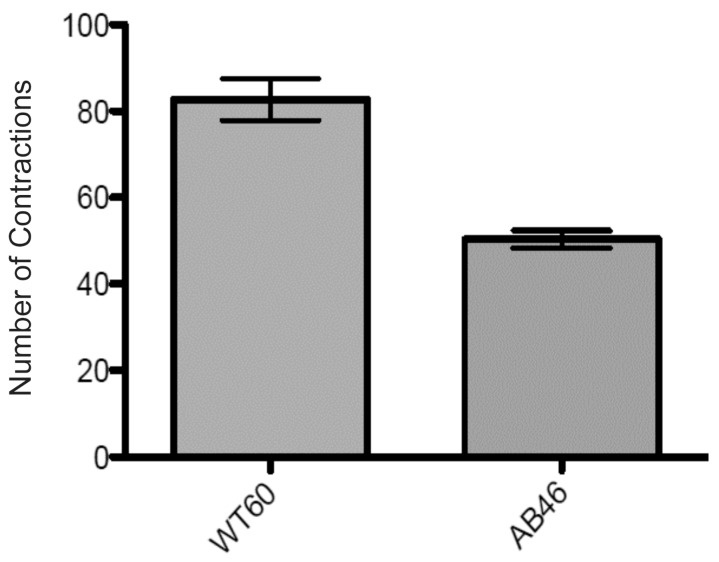
Mouth hook contraction rate. The number of larval mouth hook contractions was measured using larvae as described in the text. *p* < 0.0001 using *t*-test and 2-way ANOVA with six trials per genotype. Error bars show SEM.

**Figure 8:**
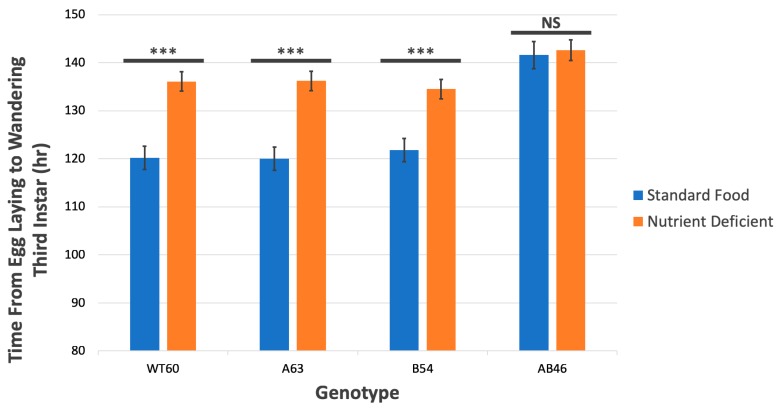
Effect of nutrient deficiency on larval development. Larvae of the indicated genotype were raised on standard food or a nutrient deficient medium (10% of the standard food). *N* = six trials of each genotype. *** indicates *p* < 0.0001. NS = not significant. Similar results were obtained with both *t*-test and one way ANOVA.

Three lines of evidence indicate that the prolonged period of third instar development of larvae lacking a wild-type allele of either *MSR* gene is due to a slower rate of food consumption. First, over a period of 5.5 h, this *MSR* double mutant (AB46) consumed 42% less food than wild-type larvae as measured by uptake of ^32^P labeled food (Figure 6). Second, the rate of mouth hook contractions was significantly reduced in larvae lacking MSR. When larvae are placed on a nutrient agar plate, they use their mouth hooks to propel themselves across the surface and this is an important part of feeding behavior [39]. Larvae lacking MSR (AB46) had a 40% slower rate of mouth hook contraction compared to wild-type (Figure 7). Third, wild-type (WT60), and the *MSR* single-mutants (A63 and B54) exhibit a delayed third instar development when reared on a nutrient deficient food (10% of the normal food content); similar to that observed with the *MSR* double-mutant (AB46) raised on standard food (Figure 8). This prolonged third instar larval developmental delay when food is limiting has been reported [40]. However, the MSR-deficient (AB46) larvae did not have any significant change in third instar development on the nutrient-deficient food (Figure 8). This indicates that for the AB46 larvae, the diminished food availability was not limiting. The single MSR deletion strains (A63 and B54) were not included in these studies since there were no significant differences between these lines and the wild-type (WT60) in any of the previous experiments.

## 4. Discussion

The knockout of both *MSRA* and *MSRB* loci in *Drosophila* provides the first characterization of an animal model system lacking all known MSR activity. Loss of all known MSR has previously been reported in bacteria [41] and yeast [30] and while these organisms provide valuable information, they are not as developmentally complex as *Drosophila*. The double MSR deletion *Drosophila* model is the most relevant MSR model system at this time, and it will be interesting to compare it to the recently created quadruple *MSR* knock-out mouse [35].

The correlation between the loss of MSRA and reduced longevity has been documented in yeast [30], *C. elegans* [29] and mice [24], although a more recent study on an *MSRA* knockout mouse did not see an effect on lifespan [42]. Almost all of the assumptions that were made on what the phenotype of the *MSR* knockout *Drosophila* would be turned out to be wrong. Based on what was known, it was predicted that the loss of either a single MSR or both MSRA and MSRB would produce a fly line with reduced longevity, elevated levels of protein oxidation, and increased sensitivity to oxidative stress. What was found is a significantly shorter lifespan in the *MSR* double mutant, but a single wild-type allele of either *MSRA* or *MSRB* restored a nearly normal lifespan (Figure 3). The shortened lifespan in the absence of any known MSR activity could not be explained by increased levels of oxidative stress (Figure 4 and Figure 5).

Direct evidence that methionine oxidation increases in the absence of MSR activity in any animal model has not been reported. The activities of all known MSR enzymes are highly stereospecific. MSRA reduces the met-S-(o) epimer while MSRB reduces the met-R-(o) epimer. Chemical oxidation is not stereospecific and we would expect that met-S-(o) would accumulate in an *MSRA* mutant (*MSRA^∆/∆^MSRB^+/+^*). Similarly, met-R-(o) would accumulate in the strains lacking *MSRB* (*MSRA^+/+^MSRB^∆/∆^*). If met-(o) levels increased significantly, we would expect some demonstrable biological effects. This may be occurring in the *MSRA* knockout studies in yeast [30], *C. elegans* [29] and mice [24] although met-(o) levels were not reported. In sharp contrast to these studies, our experiments with *Drosophila* failed to find a significant phenotype in animals lacking just one of the two *MSR* genes. Yet, in the absence of any MSR activity (*MSRA^∆/∆^MSRB^∆/∆^*), there is a significantly longer third instar where the larvae grow at a slower rate and then the adult flies have a shortened lifespan. One straightforward explanation would be that in *Drosophila*, missing one *MSR* gene leads to tolerable levels of met-(o) that can be effectively handled through the process of protein degradation, while the loss of both *MSR* genes overwhelms the cell’s protein degradation system, thus leading to the observed phenotypes. An alternative explanation would be the presence of a novel epimerase activity capable of interconverting the two met-(o) epimers. This three-component system would be capable of reducing both epimers of met-(o) as long as two parts of the system are functioning. The existence of such an epimerase for methionine sulfoxide has not been reported but has been suggested by several other investigators [43,44,45].

The results of experiments involving larvae reared on a nutritionally deficient food (Figure 8) offer an intriguing possibility of an interaction between MSR and serotonin. The wild-type, as well as the single *MSR* mutant larvae, exhibits a prolonged third instar development that is similar to the MSR-deficient larvae maintained on complete standard food. However, these MSR-deficient larvae do not show any significant effect on third instar larval development on the nutritionally compromised food. Collectively, the data indicate that the MSR-deficient larvae grow at a slower rate than wild-type or the single MSR-mutants, most likely due to a reduced rate of feeding.

The prolonged third instar larval development of wild-type larvae raised on the nutritionally deficient food has been reported previously [40]. The phenotype is remarkably similar to that of the MSR-deficient animals when reared on standard complete food. Ecdysteroid is a key hormone in the transition from larva to adult. Synthesis of this steroid hormone is regulated by a subset of serotonergic neurons that innervate the prothoracic gland, which produces the ecdysteroid. These investigators found nutritional deprivation alters the morphology of these neurons, suggesting that these serotonergic neurons form a communication link between the external environment and this internal endocrine system by affecting the timing of steroid biosynthesis. These data suggest an intriguing possibility that the absence of MSR may influence serotonin synthesis and ultimately ecdysteroid production, which in turn alters larval development.

Serotonergic neurons are responsible for a variety of processes including the coordination of feeding behaviors as well as the control of body mass [46]. Investigators have reported that an excess of serotonin causes morphological aberrations known as neuritic spheroids leading to ubiquitination and axonal degeneration by apoptosis and autophagic hindrance [47]. Morphogenesis or apoptosis of serotonergic neurons in *Drosophila* has been shown to affect muscle development and lifespan. These investigators theorized that a lack of serotonergic neurons leads to possible mutations to receptors in the insulin/insulin-like growth factor (IGF) signaling pathway extending or reducing adult longevity. Furthermore, these mutations have been found to alter downstream signaling events important for muscle development, integrity, and function during larval and adult development. These data further propose the possibility that the absence of MSR activity may influence serotonin synthesis leading to a variety of detrimental phenotypes.

## 5. Conclusions

In summary, the in vivo effects of MSR deficiency using *Drosophila* as a genetic model were evaluated. Unexpectedly, the absence of one of the two MSR activities showed no clear phenotype whereas loss of all known MSR resulted in a prolonged third instar development and a shortened lifespan for the adult. These results suggest two interesting questions. First, does *Drosophila* have an epimerase activity that interconverts the two epimers of met-(o)? Such an activity has been suggested by several different investigators but has never been reported [43,44,45]. Second, does MSR deficiency affect a subset of serotonergic neurons and ultimately modulate the synthesis of ecdysteroid?

## Figures and Tables

**Table 1:** Description of Methionine Sulfoxide Reductase (MSR) Strains.

Phenotype	Strain Designation	Genotype
**Wild-type**	WT60	*MSRA^+/+^MSRB^+/+^*
**No MSRA**	A63	*MSRA* ^Δ/Δ^ *MSRB^+/+^*
**No MSRB**	B54	*MSRA^+/+^MSRB* ^Δ/Δ^
**No MSR**	AB46	*MSRA* ^Δ/Δ^ *MSRB* ^Δ/Δ^

Alleles indicated by a Δ/Δ superscript are homozygous null deletion mutations. More detailed description of the strains is provided in the text. A detailed discussion of how the genetic strains used in this study were created and the rationale for their selection is given in the Supplementary Materials.

**Table 2:** MSR-deficiency leads to a prolonged development of third instar larvae.

Developmental Stage	*MSRA^+/+^MSRB^+/+^*	*MSRA^Δ/Δ^MSRB^+/+^*	*MSRA^+/+^MSRB^Δ/Δ^*	*MSRA^Δ/Δ^MSRB^Δ/Δ^*
First Instar	26.0 h	26.0 h	25.5 h	25.7 h
Second Instar	51.1 h	51.3 h	50.1 h	51.1 h
Third Instar	77.5 h	77.2 h	76.2 h	78.0 h
Wandering Third Instar	120.2 h	120.0 h	121.8 h	141.6 h
Pupariation	127.5 h	127.3 h	128.0 h	147.4 h
Eclosion	228.2 h	228.6 h	225.7 h	247.6 h

The strain designation is described in Table 1. The biological endpoints to determine each stage in development are described in the text. Both *t*-test and ANOVA analyses showed no significant difference at any stage of development in any pairwise combinations of strains WT60, A63 and B54. The only significant difference was the additional 20–21 h for the third larval instar of AB46 to reach the wandering third instar stage (*p* < 0.0001).

**Table 3:** Wet mass of female larvae.

Developmental Stage	*MSRA^+/+^MSRB^+/+^*	*MSRA^Δ/Δ^MSRB^+/+^*	*MSRA^+/+^MSRB^Δ/Δ^*	*MSRA^Δ/Δ^MSRB^Δ/Δ^*
72 h	0.37 mg	0.38 mg	0.37 mg	0.34 mg
96 h	1.34 mg	1.35 mg	1.35 mg	0.72 mg
Wandering Third Instar	1.87 mg	1.88 mg	1.86 mg	1.83 mg
Newly Eclosed Adult	1.22 mg	1.26 mg	1.23 mg	1.19 mg
6-Day Old Adult	1.51 mg	1.51 mg	1.57 mg	1.44 mg

The wet mass of female larvae was measured at the indicated developmental stages. The biological endpoints used to determine sex and developmental stage are described in the text. The results are the average of five trials using ten larvae for each trial. The only significant difference was found at 96 h which is the midpoint in the third larval instar development (indicated by bold text). The *p*-value for differences between WT60, A63 and B54 compared to AB46 was <0.0001.

## Supplementary Materials

The following are available online at http://www.mdpi.com/2076-3921/7/11/155/s1, Figure S1: Diagrammatic representation of the genomic deletion in *MSRA*. MSRA P-element excision between −300 and 1172 bp of the Eip71C gene on the 3L arm in a YW background. Blue segments correspond to the amino acid coding sequence of the gene, Figure S2: Longevity of *MSRA* Deletion and Wildtype Revertants. Lines 16671^w16^, 16671^w45^, 16671^w72^ are MSRA wildtype due to precise excision of the P-element, whereas 16671^w90^ is a null mutant due to ~1.4 Kbp deletion of the *MSRA* locus. The results are the average number of flies living after transfer to normal culture medium. A total of 50 flies (five vials with 10 flies each) were used for each strain. The strains used were 16671^w16^ (wildtype control ♦), 16671^w45^ (wildtype control ■), 16671^w72^ (wildtype control ▲) and 16671^w90^ (MSRA deletion ×). The error bars are standard error of the mean, Figure S3: Lifespan differences between the YW and Biocore wildtype lines. The wildtype line designated Biocore had a statistically significant decreased lifespan compared with the wildtype line “YW” (*p* < 0.001, log-rank test for survival). The maximum lifespan of the YW line was 85 days compared with 46 for the Biocore line. The median lifespan was 64 versus 39 days for each line respectively, Figure S4: Diagrammatic representation of the genomic deletion in the *MSRB* deletion allele. MSRB P-element excision between -364 and 2163 bp of the *SelR* (*MSRB*) gene on the 3R arm in a Biocore background. Again, blue segments correspond to the coding sequence of the gene, Figure S5: The Life Span of the MSRB deletion lines are similar to that of the YW Wildtype. The *MSRB* deletion lines designated 17116^w2^ and 17116^w26^ were statistically different than the YW control line (*p* = 0.003 and *p* > 0.0001, respectively, log-rank test for survival), but line 17116^w92^ was statistically identical to the YW control (*p* = 0.6186, log-rank test of survival), Figure S6: Fly Crosses. A flow chart diagramming the crosses that were involved in creating the MSR single and double deletion lines.
