# Supplementary material for: In Vivo Effects of Methionine Sulfoxide Reductase Deficiency in Drosophila melanogaster
Source: Antioxidants (Basel). 2018 Nov 1;7(11):155. doi: 10.3390/antiox7110155 (PMC6262642; doi:10.3390/antiox7110155)
Supplement: Supplementary file 1 [file antioxidants-07-00155-s001.pdf]

Supplementary Material

In vivo effects of methionine sulfoxide reductase deficiency in Drosophila melanogaster

Lindsay Bruce [1], Diana Singkornrat [1] , Kelsey Wilson [1], William Hausman [1], Kelli Robbins [1], Lingxi Huang [1] Katie Foss [1] and David Binninger [1,2]*

Creation of the MSR Genetic Knockout Strains of Drosophila

The MSRA and MSRB deletion lines were created by imprecise P-element excisions. The following is a detailed discussion of how the lines described in Table 1 of the manuscript were obtained.

Overview

1. Recovery of four independent MSRA deletion alleles
2. Recovery of three MSRB deletion alleles
3. Rationale for selecting one MSRA and MSRB allele
4. Recombination of the MSRA and MSRB lines to obtain MSRA/MSRB double deletion lines.

Creation of the MSRA deletion line

Stock 16671, which contains a P-element insertion in Exon 2 of the MSRA gene was obtained from the Bloomington Stock Center (Bloomington, IN). This line was isogenized by several generations of backcrossing to a standard YW wildtype stock line. After crosses to mobilize the P-element, four MSRA deletion mutants were recovered. Two lines had ~1.4 kb deletions (lines 16671^{w90} and 16671^{w92}) while two lines had ~1.0 kb deletions (lines 16671^{w16} and 16671^{w49}). We focused on the two larger deletions since we wanted to maximize recovery of a deletion mutation with no MSRA activity. Since both of the larger deletions were similar in size and the breakpoints in the DNA occurred at roughly the same locations, we moved ahead with line 16671^{w90}. Molecular analyses showed that most of the proximal promoter, the entire 5'UTR and a portion of the amino acid coding region had been removed (Supplemental Figure 1). RT-PCR (Figure 1 in the manuscript) and Western blotting (Figure 2 in the manuscript) failed to detect any mRNA or protein, respectively.

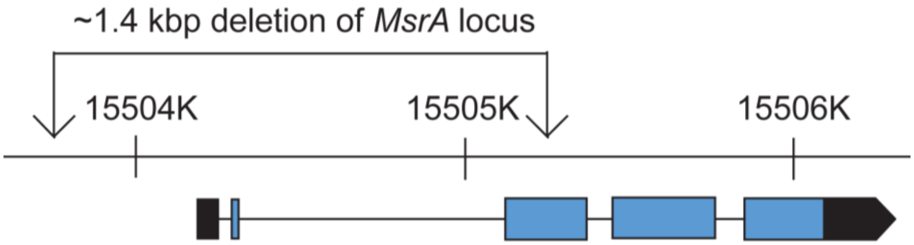

Supplemental Figure 1: Diagrammatic representation of the genomic deletion in *MSRA*. MSRA P-element excision between -300 and 1172 bp of the Eip71C gene on the 3L arm in a YW background. Blue segments correspond to the amino acid coding sequence of the gene.

Along with the four MSRA deletion lines, three wild-type revertants (16671^{w16}, 16671^{w45}, 16671^{w72}) were obtained by precise excision of the P-element. Reversion to a wild-type allele was confirmed for all three strains by DNA sequencing. Additionally, results of RT-PCR and Western blotting were indistinguishable from the wildtype control (data not shown). Lifespan assays using the 16671^{w90} strain and the three putative wild-type revertants showed no significant differences

(Supplemental Figure 2). The lifespan assay was repeated in the presence or absence of 2mM paraquat (as used in Figure 5 in the manuscript) with these four lines. There were no significant differences in the four lifespan curves (data not shown).

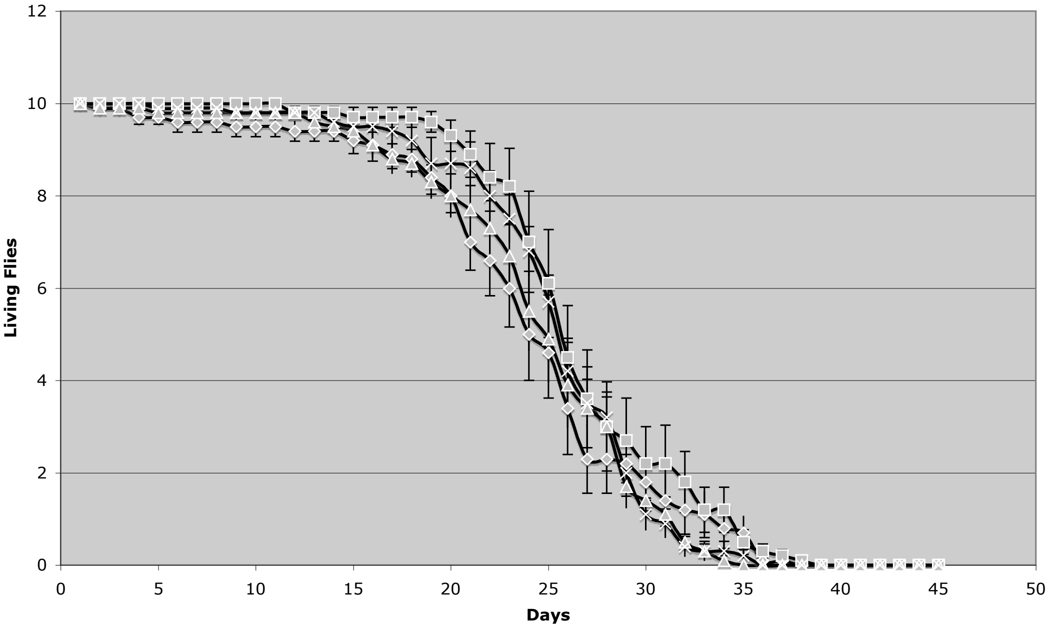

Supplemental Figure 2: Longevity of *MSRA* Deletion and Wildtype Revertants. Lines 16671^{w16}, 16671^{w45}, 16671^{w72} are MSRA wildtype due to precise excision of the P-element, whereas 16671^{w90} is a null mutant due to ~1.4 Kbp deletion of the *MSRA* locus. The results are the average number of flies living after transfer to normal culture medium. A total of 50 flies (five vials with 10 flies each) were used for each strain. The strains used were 16671^{w16} (wildtype control ◆), 16671^{w45} (wildtype control ■), 16671^{w72} (wildtype control ▲) and 16671^{w90} (MSRA deletion ✕). The error bars are standard error of the mean.

After determining there were no phenotypic differences between the MSRA deletion lines and the controls, the MSRA null line 16671^{w90} was used as one of the parental strains for creating the double MSR deletion line (described below).

Creation of the MSRB deletion line

Stock 17116 having a P-element in the MSRB locus was obtained from the Bloomington Stock Center (Bloomington, IN). The P-element was inserted in the proximal promoter region of the MSRB gene, just a few bases upstream of the transcriptional start site.

The stock was received as a heterozygous stock, having the TM6B balancer for chromosome 3 because it was homozygous lethal. Recombination with a wild-type stock designated Biocore, a white eyed wildtype line from the University of Wisconsin-Madison [1], removed the recessive lethal mutation, and the stock became fully viable when homozygous for the P-element insertion. We did not use the YW stock for this isogenization process because our YW stock line had become contaminated and we had the Biocore wildtype line available from our colleague Ken Dawson-Scully. The stock originated from the Bloomington Stock Center (Bloomington, IN; Flybase ID FBal0033148). After isogenization with Biocore, the 17116 P-element line exhibited a small, but detectable amount of MSRB mRNA using RT-PCR. Anecdotally, the line still seemed not as "robust" as the YW wildtype

lines. We felt this might be due, at least in part, to the dramatically shorter lifespan of the Biocore line compared to the YW wildtype strain, which we obtained a second time from the Rodney Murphey lab (FAU, Boca Raton, FL) since our original stock, as noted previously, had become contaminated (Supplemental Figure 3). The 17116 stock was backcrossed to the newly acquired YW line for several generations.

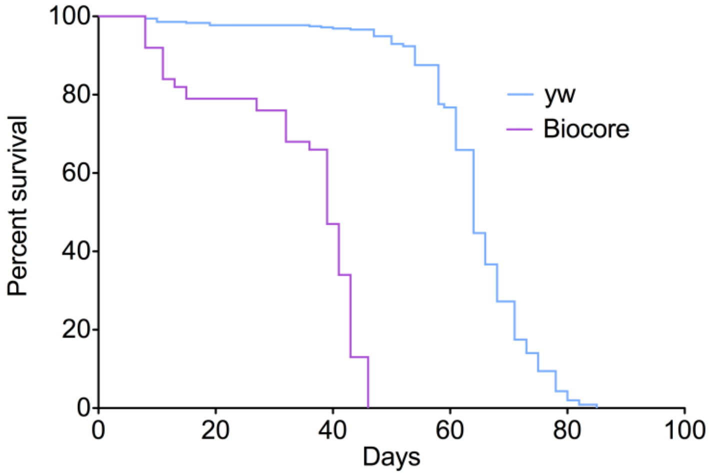

Supplemental Figure 3: Lifespan differences between the YW and Biocore wildtype lines. The wildtype line designated Biocore had a statistically significant decreased lifespan compared with the wildtype line "YW" ($p < 0.001$, log-rank test for survival). The maximum lifespan of the YW line was 85 days compared with 46 for the Biocore line. The median lifespan was 64 versus 39 days for each line respectively.

Using the same methodology employed in the creation of the *MSRA* deletion lines, the YW isogenized 17116 line was exposed to transposase to create an *MSRB* deletion line. We recovered three lines containing a ~2.5 kb deletion in *MSRB* extending from a site just upstream of the transcriptional start site into the third exon, which is shared by all isoforms (Supplemental Figure 4). Homozygous lines for each of these three *MSRB* deletions showed no detectable transcript or protein using RT-PCR and Western blotting, respectively (data not shown).

The three *MSRB* deletion lines (17116^{w2}, 17116^{w26}, and 17716^{w92}) were compared to the recovered YW wildtype line in a lifespan assay. The survival curves were similar to one another with the median survival for all the lines between 37.5 and 43 days, but line 17716^{w92} had a survival curve that was statistically indistinguishable from the YW wildtype line and was selected to use in creating the MSR double deletion line described below (Supplemental Figure 5).

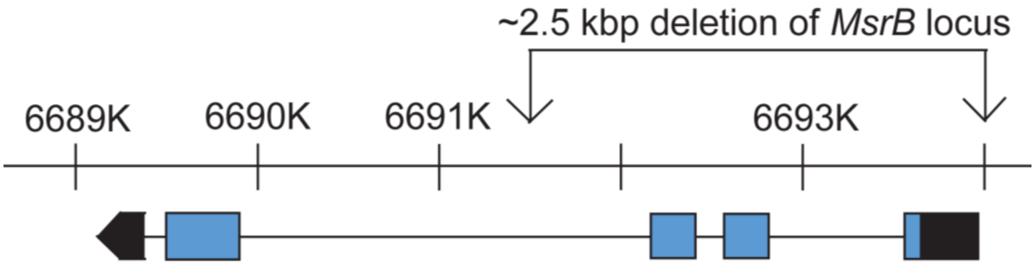

Supplemental Figure 4: Diagrammatic representation of the genomic deletion in the *MSRB* deletion allele. MSRB P-element excision between -364 and 2163 bp of the *SelR* (*MSRB*) gene

on the 3R arm in a Biocore background. Again, blue segments correspond to the coding sequence of the gene.

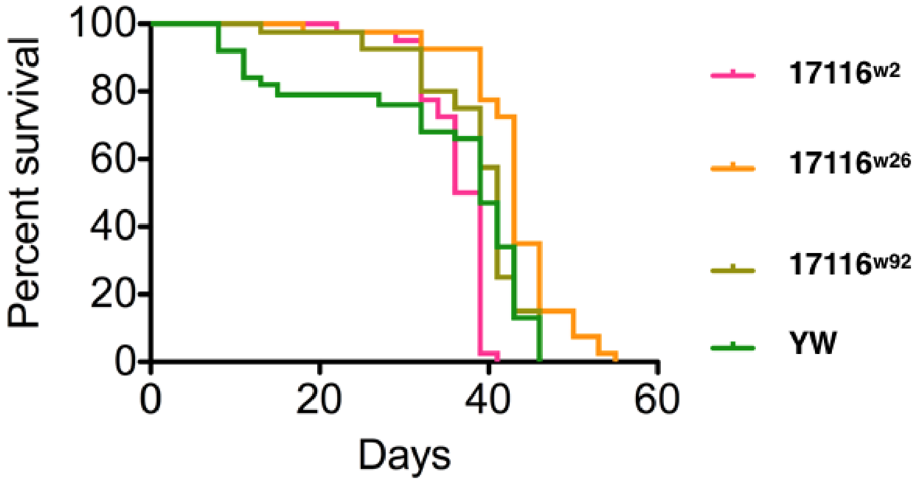

Supplemental Figure 5: The Life Span of the MSRB deletion lines are similar to that of the YW Wildtype. The *MSRB* deletion lines designated 17116^{w2} and 17116^{w26} were statistically different than the YW control line ($p = 0.003$ and $p > 0.0001$, respectively, log-rank test for survival), but line 17116^{w92} was statistically identical to the YW control ($p = 0.6186$, log-rank test of survival).

Creation of the MSR Double Deletion Line and Molecular Analysis

An MSRA$^{\Delta/\Delta}$MSRB$^{\Delta/\Delta}$ line was created by recombining the MSRA$^{\Delta/\Delta}$ line (16671^{w90}) with the MSRB$^{\Delta/\Delta}$ line (17116^{w92}). Due to concern over the different survival phenotypes of the MSRB$^{\Delta/\Delta}$ lines compared to the MSRA$^{\Delta/\Delta}$ line being primarily due to differences in wildtype background, MSRA$^{\Delta/\Delta}$MSRB$^{\Delta/\Delta}$ line was isogenized and then recombined with the newly obtained YW strain from the Rodney Murphey lab at FAU. From this cross 100 independent sibling lines were created with four genotypes resulting: *MSRA$^{+/+}$MSRB$^{+/+}$*, *MSRA$^{\Delta/\Delta}$MSRB$^{+/+}$*, *MSRA$^{+/+}$MSRB$^{\Delta/\Delta}$*, and MSRA$^{\Delta/\Delta}$MSRB$^{\Delta/\Delta}$. Of these 100 lines, 11 lines were used for the experiments described hereafter. These 11 lines included four wildtype control lines (WT11, WT31, WT59, and WT60), three *MSRA* deletion lines (A44, A63, and A90 – a separate line from the 16671^{w90} *MSRA* deletion line), one *MSRB* deletion line (B54), and three *MSR* double deletion lines (AB16, 21, and 46). All other lines besides the single *MSRB* deletion line were tested in triplicate (or greater in the case of the wildtype controls). A summary of the creation of the lines is shown in Supplemental Figure 6.

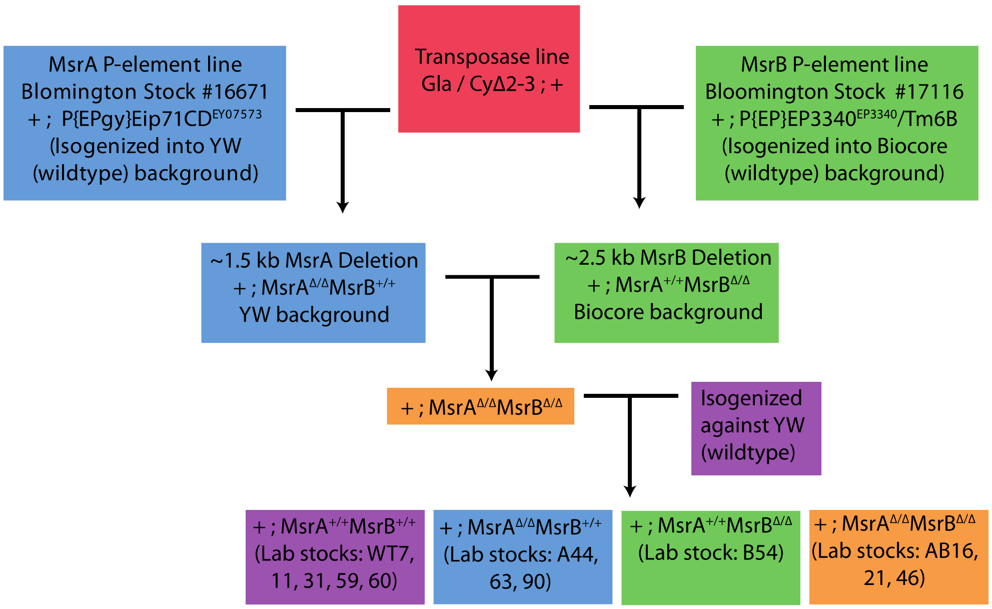

Supplemental Figure 6: Fly Crosses. A flow chart diagramming the crosses that were involved in creating the MSR single and double deletion lines.

Initially, all eleven lines were used for the lifespan, oxidative stress, and protein oxidation assays. After determining that the various lines behaved remarkably similarly by genotype, further assays (larval development and nutrient deficiency) were completed with only one line from each genotype – WT60, A63, B54 and AB46. After no differences were shown in the larval development assay between the single *MSR* mutants and the wildtype line, only the double *MSR* mutant (AB46) and the wildtype line (WT60) were used for the remaining mouth hook contraction and food consumption assays as there was no need to explain a phenotype for the single *MSR* mutants.

References:

1. Phillis, R.W.; Bramlage, A.T.; Wotus, C.; Whittaker, A.; Gramates, L.S.; Seppala, D.; Farahanchi, F.; Caruccio, P.; Murphey, R.K. Isolation of Mutations Affecting Neural Circuitry Required for Grooming Behavior in Drosophila Melanogaster. *Genetics*. **1993,** 133(3): 581-592.
